# Transcriptome Analysis of *Cordyceps militaris* Reveals Genes Associated With Carotenoid Synthesis and Identification of the Function of the *Cmtns* Gene

**DOI:** 10.3389/fmicb.2019.02105

**Published:** 2019-09-10

**Authors:** Hai-Wei Lou, Yu Zhao, Hong-Biao Tang, Zhi-Wei Ye, Tao Wei, Jun-Fang Lin, Li-Qiong Guo

**Affiliations:** ^1^Department of Bioengineering, College of Food Science, South China Agricultural University, Guangzhou, China; ^2^College of Food Science and Technology, Henan University of Technology, Zhengzhou, China; ^3^Research Center for Micro-Ecological Agent Engineering and Technology of Guangdong Province, Guangzhou, China

**Keywords:** *Cordyceps militaris*, transcriptome, pigment, illumination, carotenoid, terpenoid synthase

## Abstract

*Cordyceps militaris*, a valuable edible and medicinal fungus, has attracted increasing attention because of its various bioactive ingredients. However, the biosynthetic pathway of *C. militaris* carotenoids is still unknown due to lack of transcriptome information. To uncover genes related to the biosynthesis of *C. militaris* carotenoids, the transcriptomes of mycelia CM10_D cultured under dark conditions and mycelia CM10_L cultured under light exposure conditions were sequenced. Compared with mycelia CM10_D, 866 up-regulated genes and 856 down-regulated genes were found in mycelia CM10_L. Gene ontology (GO) analysis of differentially expressed genes (DEGs) indicated that DEGs were mainly classified into the “metabolic process,” “membrane,” and “catalytic activity” terms. Kyoto Encyclopedia of Genes and Genomes (KEGG) pathway enrichment analysis of DEGs suggested that DEGs were mainly enriched in “metabolic pathways,” “MAPK signaling pathway-yeast,” and “biosynthesis of secondary metabolites.” In addition, the carotenoid content of the *Cmtns* gene deletion mutant (Δ*Cmtns*) was significantly lower than that of the wild-type *C. militaris* CM10, while the carotenoid content of the complementary strain (Δ*Cmtns-c*) of the *Cmtns* gene was not significantly different from that of *C. militaris* CM10, suggesting that the *Cmtns* gene significantly affected the biosynthesis of carotenoids in *C. militaris*. These results potentially pave the way for revealing the biosynthetic pathway of carotenoids and improving carotenoids production in *C. militaris*.

## Introduction

*Cordyceps militaris*, as a model species of *Cordyceps* fungi, is widely used as a nourishing food and used in traditional Chinese medicine in East Asia (Zhang et al., 2018; Kunhorm et al., 2019). *C. militaris* is also often used as a substitute for *Ophiocordyceps sinensis* because it contains similar bioactive components (including cordycepic acid, polysaccharides, adenosine, ergosterol, etc.) (Zhu et al., 2016; Wang et al., 2017; Yin et al., 2018). It is noteworthy that *C. militaris* contains cordycepin and pentostatin which *O. sinensis* does not contain (Xia et al., 2017). These bioactive ingredients endow *C. militaris* with anti-inflammatory, anti-tumor, anti-cancer, anti-oxidation, and hypoglycemic activities (Zhang et al., 2006; Lee et al., 2015; Kim et al., 2017; Hu et al., 2019; Liu et al., 2019). Based on this, the market demand of *C. militaris* is increasing year by year.

Fungi often produce a variety of pigments that give them different color phenotypes (Lu et al., 2013). Similarly, *C. militaris* produces pigments that make it yellow or orange (Yang et al., 2016). The pigments contained in *C. militaris* were first considered to be carotenoids and water-soluble (Friederichsen and Engel, 1958). In 2013, four novel water-soluble carotenoids were isolated and identified from *C. militaris* (Dong et al., 2013b). Traditional carotenoids are fat-soluble pigments and insoluble in water, while *C. militaris* carotenoids can be dissolved in water, which will expand the application field of *C. militaris*. However, the content of carotenoids in *C. militaris* is low, which cannot meet the market demand. Up to now, the biosynthetic pathway of *C. militaris* carotenoids is still unknown, and there are few reports on the related genes of carotenoid biosynthesis. Therefore, it is urgent to study the biosynthetic pathway and related genes of *C. militaris* carotenoids in order to improve the content of carotenoids by genetic engineering.

Light is an essential environmental factor for the production of carotenoids in *C. militaris*. After exposure to light, the colony color of *C. militaris* changes from white to yellow or orange (Yang et al., 2016; Sun et al., 2017; Zhang et al., 2017). This indicates that light can induce the expression of genes synthesizing *C. militaris* carotenoids. Therefore, the gene expression profiles of *C. militaris* cultured under different light exposure conditions should be different. RNA sequencing (RNA-seq) technology is an efficient, rapid, and sensitive method to deeply study and analyze biological transcriptomes, which provides a whole perspective for the study of gene expression at RNA levels, whether low-expression genes or differentially expressed genes (DEGs; Zhao et al., 2019). Based on this, the aim of this study was to identify genes involved in the synthesis of carotenoids and the biosynthetic pathway of carotenoids by comparing the transcriptomes of two *C. militaris* mycelial samples cultured under different light exposure conditions. To further validate transcriptome data, gene expression analysis using quantitative real-time PCR analysis (qRT-PCR) was performed. In addition, the terpenoid synthase gene (*Cmtns*) of *C. militaris* was complemented to its deleted mutant (Δ*Cmtns*) to identify the function of the *Cmtns* gene. This study provides a better understanding of the biosynthetic mechanism of carotenoids in *C. militaris* under light exposure.

## Materials and Methods

### Strains and Vectors

A laboratory and commercial strain of *C. militaris*, CM10 (Ningyang County Haixin Biological Technology Co., Ltd., Shandong, China), was used in this study. *Agrobacterium tumefaciens* strain AGL-1 (purchased from Shanghai Weidi Biotechnology Co., Ltd., Shanghai, China) carrying the vector pCAMBIA0390-Ben-Comtns was used to perform *Agrobacterium tumefaciens*-mediated transformation (ATMT) of the Δ*Cmtns* strain to achieve complementation of the *Cmtns* gene. The vector pCAMBIA0390-Ben-Comtns, containing a benomyl (*ben*) resistant gene cassette and a *Cmtns* gene (GenBank: NW_006271971.1) expression cassette, was constructed based on pCAMBIA0390-Ben in our laboratory (Supplementary Figure S1).

### Preparation of Mycelial Samples for Transcriptome Sequencing

*Cordyceps militaris* strain CM10 was inoculated on potato dextrose agar (PDA, 200.0 g potato, 10.0 g dextrose, 3.0 g KH_2_PO_4_, 1.0 g MgSO_4_, 20.0 g agar, 1000 mL distilled water) plates for activation and cultured at 25°C for 28 days (including 21 days of cultivation under dark conditions in the first phase and 7 days of cultivation under light exposure in the second phase). The CM10 strain activated on the PDA medium was cut into pieces of 1 cm × 1 cm size, and three pieces were inoculated into 100 mL of potato dextrose broth (PDB, 200.0 g potato, 10.0 g dextrose, 3.0 g KH_2_PO_4_, 1.0 g MgSO_4_, 1000 mL distilled water), and cultured at 25°C on a shaker at 150 rpm for 4 days in the dark. 5 mL PDB culture was inoculated into solid medium (14.6 g wheat powder, 4.0 g wheat bran powder, 1.4 g corn powder, 25 mg MgSO_4_, 25 mg KH_2_PO_4_, 1 mg vitamin B_1_, 25 mL distilled water) and cultured at 25°C. The mycelial sample CM10_D was obtained after 18 days of cultivation under dark conditions. The mycelial sample CM10_L was obtained by first culturing for 10 days under dark conditions and then culturing for 8 days under light exposure conditions (750 Lux). These two mycelial samples cultured under different light exposure conditions were frozen in liquid nitrogen and immediately stored in a −80°C freezer for RNA extraction.

### cDNA Library Construction and Transcriptome Sequencing

The *C. militaris* mycelia CM10_D from six biologically repeated petri dishes were mixed together to obtain the mycelial sample CM10_D. Similarly, the *C. militaris* mycelia CM10_L from six biologically repeated petri dishes were mixed together to obtain the mycelial sample CM10_L. The total RNA was extracted from mycelial samples using TRIzol reagent (Invitrogen, Carlsbad, CA, United States) according to the manufacturer’s instructions. RNA quality and concentration were evaluated using an Agilent 2100 bioanalyzer (Agilent Technologies, Santa Clara, CA, United States) and NanoDrop Spectrophotometer (NanoDrop Technologies, Wilmington, DE, United States). The mRNA of each sample was then isolated according to the polyA selection method using oligo (dT) beads. Two cDNA libraries were generated using a NEB Next Ultra RNA Library Prep Kit for Illumina (NEB, Ipswich, MA, United States) following the manufacturer’s recommendations. Each cDNA library was sequenced once from both ends on an Illumina HiSeq 4000 platform at Beijing Genomics Institute (BGI, Shenzhen, China). Based on the sequencing quality assessment, ambiguous reads that had more than 5% unknown bases (“N”) or a sequencing quality was less than 20% per read were filtered out using in-house software developed by BGI. The remaining reads with a length of 100 base pairs (bps), called clean reads, were then proceeded through the Trinity reconstruction pipeline to generate full-length transcripts (Haas et al., 2013). All subsequent analyses in this study were based on the clean data. The clean data were deposited in NCBI Sequence Read Archive (SRA) under the BioProject accession number PRJNA541819.

### Genome Mapping and Analysis of Differentially Expressed Genes

The clean reads were mapped to the *C. militaris* genomic data (BioProject accession no. PRJNA225510) using HISAT (v0.1.6) (Zheng P. et al., 2011; Kim et al., 2015). The number of reads mapped to genes was normalized for RNA length according to FPKM (fragments per kilobase of exon per million mapped reads). After mapping to the reference genome, we used StringTie to reconstruct transcripts (Pertea et al., 2015), and with genome annotation information we could identify novel transcripts exist in our samples use cuffcompare, a tool of cufflinks (Trapnell et al., 2012). After novel transcript detection, we merged novel coding transcripts with reference transcripts to get the complete reference, then we mapped clean reads to it use Bowtie2 (Langmead and Salzberg, 2012) and calculated gene expression level for each sample with RSEM (Li and Dewey, 2011).

Gene expression levels were normalized using the FPKM method. Differential expression analysis was performed using the Deseq2 tool (Love et al., 2014). The *p*-values were adjusted using the method of Benjamini and Hochberg (1995). Unigenes with a false discovery rate (FDR) ≤0.001 and a fold change ≥2 in a comparison (CM10_D vs CM10_L) were identified as significantly DEGs.

### Functional Annotation of DEGs

For functional annotation and analysis, all DEGs derived from RNA-seq data were annotated by searching against the NCBI non-redundant protein sequence database (NR), gene ontology (GO) database^1^, and Kyoto Encyclopedia of Genes and Genomes (KEGG) database^2^ (Mao et al., 2005; Young et al., 2010). Furthermore, the annotated data of *C. militaris* strain CM01 were also used for functional annotation by BLASTN and BLASTX, respectively (Zheng P. et al., 2011).

### Validation of Transcriptome Data by Quantitative Real-Time PCR Analysis

To validate the quality of transcriptome data, five genes (CCM_04119, CCM_05246, CCM_06728, CCM_08263, and CCM_09155) were selected for qRT-PCR analysis using the elongation factor 1-alpha (*tef1*) gene (GenBank: DQ070019) as the internal control gene (Lian et al., 2014b). Total RNA of *C. militaris* mycelial samples was extracted using the Fungal RNA Kit (Omega, Stamford, CT, United States), and then reverse-transcribed using TransScript All-in-One First-Strand cDNA Synthesis SuperMix (TransGen Biotech, Beijing, China). qRT-PCR was performed using TransStart Tip Green qPCR SuperMix (TransGen Biotech, Beijing, China) with a QuantStudio 3 Real-Time PCR System (Thermo Fisher Scientific, Waltham, MA, United States). The reaction system and reaction conditions of the qRT-PCR were performed as previously described (Chen et al., 2018). The relative expression levels of genes were calculated using the 2^–ΔΔ*CT*^ method (Livak and Schmittgen, 2001). All the primers used in this study are shown in Supplementary Table S1.

### Complementation of the *Cmtns* Disruption Mutant

The only terpenoid synthase gene *Cmtns* in DEGs was obtained by transcriptome analysis. In order to study the mechanism of carotenoid biosynthesis in *C. militaris*, we performed functional identification of the *Cmtns* gene. In our previous studies, we had knocked out the *Cmtns* gene by homologous recombination and obtained the target gene mutant Δ*Cmtns* (Lou et al., 2018). In this study, the *Cmtns* gene was complemented to the Δ*Cmtns* mutant by the ATMT method. The entire *Cmtns* gene with a 2290-bp upstream region as its putative promoter (*Ptns*) and a 520-bp downstream region as the putative terminator (*Ttns*) was amplified from the *C. militaris* genome with primers CmtnsC-F/R and inserted into the binary vector pCAMBIA0390-Ben to generate the binary vector pCAMBIA0390-Ben-Comtns (Supplementary Figure S1) for *C. militaris* transformantion. The binary vector pCAMBIA0390-Ben-Comtns was transformed into *A. tumefaciens* strain AGL-1 to obtain the strain AGL1-pCAMBIA0390-Ben-Comtns. The *A. tumefaciens* strain AGL1-pCAMBIA0390-Ben-Comtns and conidia of the Δ*Cmtns* mutant were co-cultured to achieve complementation of the *Cmtns* gene by the previously described ATMT method (Zheng Z. et al., 2011). Transformants were selected on PDA plates supplemented with 3 μg/mL of benomyl at 25°C. Positive complemented transformants (Δ*Cmtns-c*) were confirmed by PCR and qRT-PCR.

### Determination of Carotenoid Content

The fruiting bodies of all strains (wild-type strain CM10, Δ*Cmtns* strain, and Δ*Cmtns-c* strain) were cultured on rice medium according to the method described in previous report (Jiang and Han, 2015). The fruiting bodies of *C. militaris* were vacuum freeze-dried, and then dried fruiting bodies were used for the determination of carotenoid content according to previously reported methods (Yang et al., 2014; Zhang et al., 2018).

### Statistical Analysis

The qRT-PCR experiment and the determination of carotenoid content were performed in triplicate. Data were analyzed by SPSS 20.0 software (SPSS Inc., Chicago, IL, United States). One-way analysis of variance (ANOVA) and Duncan’s multiple-range tests were used to determine significant differences. The values were shown as the mean ± standard error (SE). *P*-values less than 0.05 were considered significant.

## Results

### Preparation of *C. militaris* Mycelia and Summary of RNA-Seq Data Sets

**FIGURE 1:**
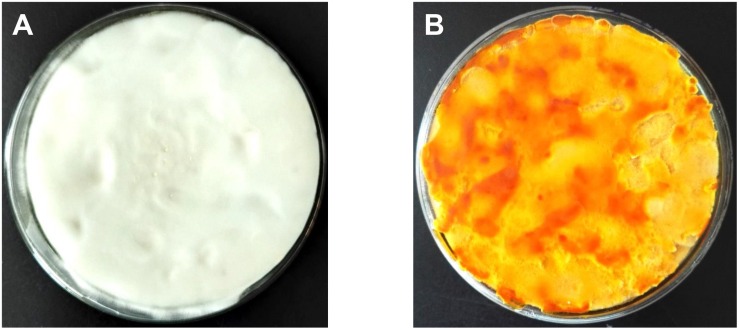
*Cordyceps militaris* mycelial samples CM10_D **(A)** and CM10_L **(B)**.

Mycelial samples of *C. militaris* cultured under different light exposure conditions are shown in Figure 1. The mycelia (CM10_D) of *C. militaris* cultured under dark conditions was pure white, and the mycelia (CM10_L) of *C. militaris* cultured under light exposure conditions was orange. It can be concluded that light is an essential factor for the production of *C. militaris* pigments.

To compare the gene expression profiles of the white mycelia and the orange mycelia, the cDNA libraries were built from cultivated mycelia (CM10_D and CM10_L) and subjected to Illumina deep sequencing. As shown in Table 1, mycelia CM10_D and mycelia CM10_L produced raw reads of 30,769,028 reads and 32,387,910 reads, respectively. After quality filtering, 29,635,460 clean reads were obtained from mycelia CM10_D and 30,261,506 clean reads were obtained from mycelia CM10_L. The Q30 values of each sample were more than 94%, indicating that the sequence data was accurate. 84.70% of CM10_D reads and 79.94% of CM10_L reads could be mapped to the *C. militaris* genome with 9684 protein-coding genes (Zheng P. et al., 2011). In addition, 8793 genes (including 8655 known genes and 138 novel genes) were detected from CM10_D and 8807 genes (including 8670 known genes and 137 novel genes) were detected from CM10_L (Supplementary Table S2).

**TABLE 1:** Summary of transcriptome sequencing.

**Sample**	**Total raw reads (Mb)**	**Total clean reads (Mb)**	**Total clean bases (Gb)**	**Clean reads Q30 (%)**	**Genome mapping ratio (%)**
CM10_D	30.77	29.64	4.45	94.71	84.70
CM10_L	32.39	30.26	4.54	94.77	79.94

### Functional Annotation and Analysis of DEGs

Transcriptome data were used to identify genes showing significant changes in expression of *C. militaris* cultured under different light exposure conditions (CM10_D and CM10_L). Compared with CM10_D, 1722 DEGs were identified in CM10_L (866 up-regulated DEGs and 856 down-regulated DEGs).

**FIGURE 2:**
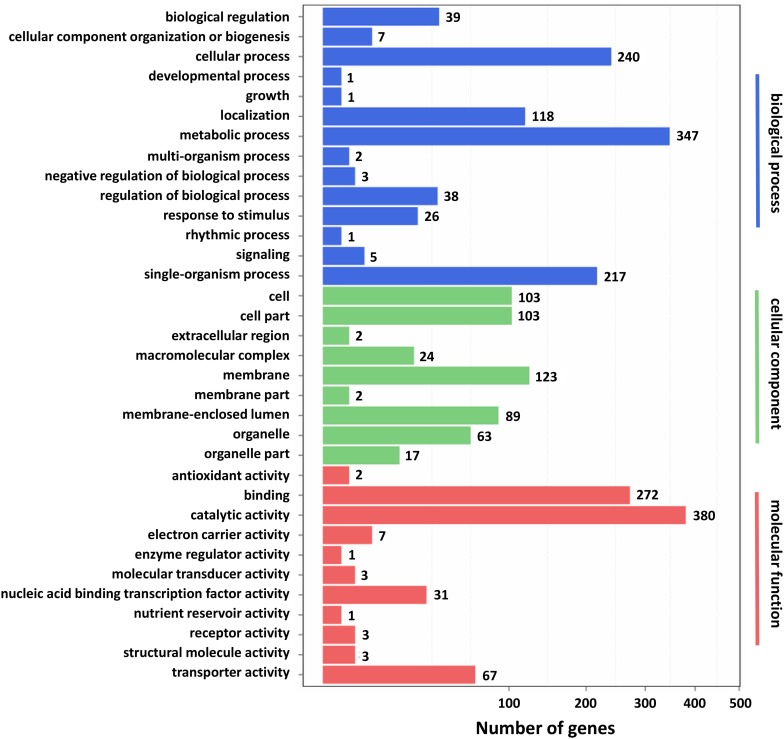
Gene ontology (GO) classification of differentially expressed genes (DEGs). *X* axis represents the number of DEGs. *Y* axis represents the GO term.

**FIGURE 3:**
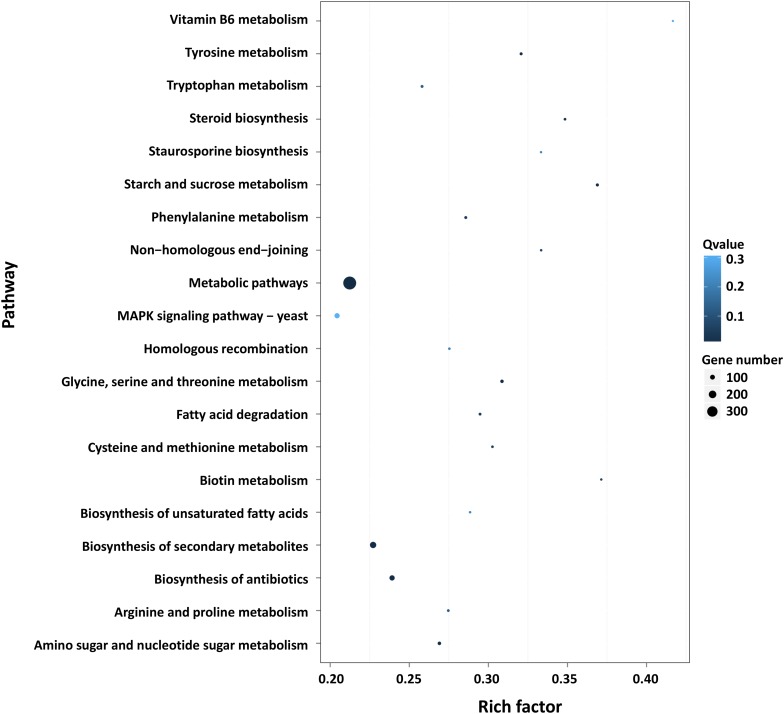
Pathway functional enrichment of DEGs. *X* axis represents the enrichment factor. *Y* axis represents the pathway name. Coloring indicates the *q*-value (high: white; low: blue), and the lower *q*-value indicates the more significant enriched. The point size indicates the number of DEGs (more: big; less: small).

Furthermore, GO and KEGG pathway analysis were carried out to study the functions of DEGs. All DEGs were mapped to the GO terms in three main categories of GO database. The GO classification results showed that 2341 DEGs were categorized into 34 GO terms (Figure 2). In the “biological process” category, it is worth noting that the term with the most DEGs was the metabolic process. In the “cellular component” category, it is clear that the term with the most DEGs was the membrane. In the “molecular function” category, it is obvious that the term with most DEGs was the catalytic activity. Therefore, DEGs classified as the “metabolic process,” “membrane,” and “catalytic activity” terms may be responsible for the biosynthesis of *C. militaris* pigments. Different genes often cooperate to play roles in different biological processes. KEGG pathway enrichment analysis helps us to understand more about the biological functions of genes (Zhao et al., 2019). The results suggested that the DEGs were specifically located in the pathways of “metabolic pathways,” “MAPK signaling pathway-yeast,” and “biosynthesis of secondary metabolites,” which indicated that these DEGs were mainly involved in cell metabolism and signal transduction (Figure 3).

It has been reported that *C. militaris* pigments were carotenoids (Dong et al., 2013a; Yi et al., 2014). Therefore, all unigenes derived from RNA-seq data were searched against the carotenoid biosynthetic pathway in the KEGG database, and the results showed that only the CCM_06728 (CAO-2) and CCM_09155 (YLO-1) genes were found to be involved in the biosynthesis of carotenoids (Supplementary Figure S2). Unexpectedly, the expression levels of these two genes (CCM_06728 and CCM_09155) were not significantly different between mycelia CM10_D and mycelia CM10_L. Because carotenoids belong to terpenoids, the only terpenoid synthase gene *Cmtns* in DEGs was significantly up-regulated. We believe that the *Cmtns* gene might be involved in the biosynthesis of carotenoids in *C. militaris*.

### Validation of Transcriptome Data by qRT-PCR

To validate the transcriptome data, five genes were selected to analyze their expression levels by qRT-PCR. With mycelia CM10_D as a control sample, CCM_04119 (*Cmtns*), CCM_05246, and CCM_08263 genes were significantly up-regulated in mycelia CM10_L, while CCM_06728 and CCM_09155 genes were not significantly changed (Table 2). The results of qRT-PCR revealed that the expression levels of the five genes were consistent with the results of RNA-seq, indicating that the transcriptome data were accurate.

**TABLE 2:** The relative expression levels of five genes by qRT-PCR.

**Gene ID**	**Function**	**log2FoldChange (CM10_L/CM10_D)**	Relative expression level*(fold)	**Up/down-regulation**
CCM_04119 (*Cmtns*)	Terpenoid synthase	1.62	13.19 ± 2.41	Up
CCM_05246	Cytochrome c oxidase assembly protein subunit	3.28	11.17 ± 2.09	Up
CCM_06728	Torulene dioxygenase	0.55	1.84 ± 0.70	No significant difference
CCM_08263	Cytochrome P450 monooxygenase	3.35	13.41 ± 2.85	Up
CCM_09155	Beta-apo-4′-carotenal oxygenase	0.07	1.56 ± 0.38	No significant difference

*^∗^In the qRT-PCR experiment, the expression level of genes in the sample CM10_D was used as a control to calculate the relative expression level (fold) of genes in the sample CM10_L.*

### Complementation of the Δ*Cmtns* Mutant

**FIGURE 4:**
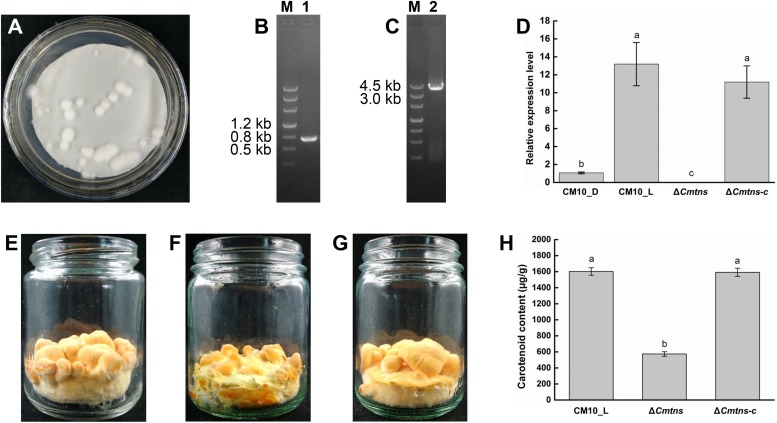
Study on the function of the *Cmtns* gene. **(A)** The conidia of the Δ*Cmtns* mutant were co-cultured with the *Agrobacterium tumefaciens* strain AGL1-pCAMBIA0390-Ben-Comtns on PDA medium supplemented with 3 μg/mL of benomyl, and the co-culture plate for complementation of the *Cmtns* gene was obtained. **(B)** PCR amplification of the *ben* gene in the Δ*Cmtns-c* strain. **(C)** PCR amplification of the *Cmtns* gene in the Δ*Cmtns-c* strain. **(D)** qRT-PCR analysis of the *Cmtns* gene expression. **(E)** *C. militaris* CM10 cultured on rice medium. **(F)** *C. militaris* Δ*Cmtns* cultured on rice medium. **(G)** *C. militaris*Δ*Cmtns-c* cultured on rice medium. **(H)** Analysis of carotenoid content in *C. militaris*. Data are means ± standard error from three independent experiments. Different letters (a–c) above the bars indicated significant difference (ANOVA followed by Duncan’s multiple-range tests, *P* < 0.05).

To identify the function of the *Cmtns* gene, the complementary experiment of the *Cmtns* gene was carried out. The co-cultured PDA plate (containing 3 μg/mL of benomyl) of complementary the *Cmtns* gene by ATMT method was shown in Figure 4A, and some resistant transformants clearly appeared on the resistant plate. Primer pairs of JCBen-F/JCBen-R and CmtnsC-F/CmtnsC-R were used to amplify the *ben* gene and the *Cmtns* gene in transformants, respectively (Figures 4B,C). The PCR products were sequenced and confirmed that the *ben* gene and the *Cmtns* cassette were successfully integrated into the genome of the Δ*Cmtns* mutant. The results of qRT-PCR analysis demonstrated that the expression of the *Cmtns* gene in mycelia CM10_L was significantly up-regulated compared with mycelia CM10_D. However, no expression of the *Cmtns* gene was detected in the Δ*Cmtns* mutant, which might be due to the deletion of the *Cmtns* gene. When the *Cmtns* gene was complemented to the Δ*Cmtns* mutant, there was no significant difference in the expression level of the *Cmtns* gene between the Δ*Cmtns-c* strain and the wild-type strain (CM10), which indicated that the *Cmtns* gene was successfully complemented to the Δ*Cmtns* mutant and could be expressed in the Δ*Cmtns-c* strain (Figure 4D).

### Effect of the *Cmtns* Gene on Carotenoid Content of *C. militaris*

The color phenotypes of the fruiting bodies of all *C. militaris* strains (CM10, Δ*Cmtns*, Δ*Cmtns-c*) were orange (Figures 4E–G). The carotenoid content of *C. militaris* Δ*Cmtns* was significantly lower than that of *C. militaris* CM10, which might be attributed to the knockout of the *Cmtns* gene. However, when the *Cmtns* gene was complemented to the Δ*Cmtns* mutant, the carotenoid content of the complementary strain Δ*Cmtns-c* was not significantly different from that of wild-type *C. militaris* strain CM10, which indicated that the *Cmtns* gene significantly affected the biosynthesis of carotenoids in *C. militaris* (Figure 4H).

## Discussion

*Cordyceps militaris* contains a variety of secondary metabolites which are beneficial to human body, and water-soluble carotenoids in *C. militaris* have attracted more and more attention (Dong et al., 2013b). Light is a necessary condition for the production of carotenoids in *C. militaris* (Yang et al., 2016). To identify the genes involved in the biosynthesis of carotenoids in *C. militaris*, the transcriptome differences between mycelia CM10_D cultured under dark conditions and mycelia CM10_L cultured under light exposure conditions were compared and analyzed. Moreover, the function of the up-regulated gene *Cmtns* was identified and significantly affected the biosynthesis of carotenoids in *C. militaris*.

There were 1722 DEGs (866 up-regulated DEGs and 856 down-regulated DEGs) between mycelia CM10_D and mycelia CM10_L. Yang et al. (2016) found that there were 1042 DEGs (545 up-regulated DEGs and 497 down-regulated DEGs) between mycelia Wt_L cultured under light exposure conditions and mycelia Wt_D cultured under dark conditions. Wang et al. (2018) found that there were only 697 DEGs (471 up-regulated DEGs and 226 down-regulated DEGs) between mycelia 498L cultured under light exposure conditions and mycelia 498D cultured under dark conditions. The number of their DEGs was significantly less than the number of DEGs in this study, which might be due to different culture conditions, different strains, and different media.

**FIGURE 5:**
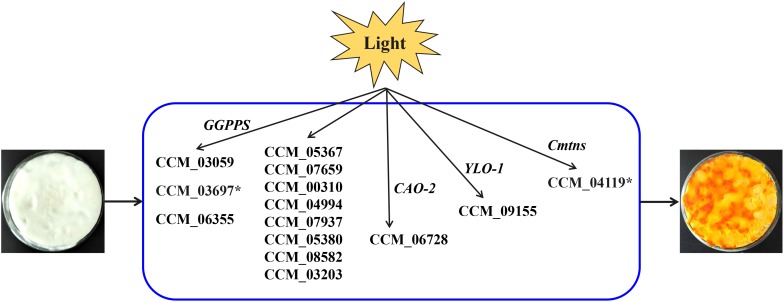
Putative model for light-induced carotenoid formation in *C. militaris* based on transcriptome analysis and previous studies. ^∗^Indicates that the gene is up-regulated in a comparison (CM10_D vs CM10_L).

*Cordyceps militaris* pigments are secondary metabolites induced by light and were considered to be carotenoids (Wang et al., 2017). However, the KEGG pathway analysis of this study indicated that only two genes (CCM_06728 and CCM_09155) were involved in the biosynthesis of carotenoids in *C. militaris*, and the expression levels of these two genes were not significantly different between mycelia CM10_D and mycelia CM10_L, which was consistent with previous studies (Yang et al., 2016; Wang et al., 2018). Three types of geranylgeranyl diphosphate synthase (GGPPS) genes (CCM_03059, CCM_03697, and CCM_06355) had been cloned from *C. militaris*, and CCM_03059 was thought to be involved in the biosynthesis of carotenoids (Lian et al., 2014a; Figure 5). In this study, the expression level of CCM_03059 gene was not significantly different between mycelia CM10_D and mycelia CM10_L, which was consistent with previous studies (Yang et al., 2016). Therefore, the CCM_03059 gene may not be directly involved in the biosynthesis of carotenoids in *C. militaris*. Nevertheless, the expression level of the CCM_03697 gene in mycelia CM10_L was significantly higher than that in mycelia CM10_D, so we believe that the CCM_03697 gene is probably responsible for the biosynthesis of carotenoids in *C. militaris*, which is different from previous studies (Lian et al., 2014a; Yang et al., 2016; Figure 5). In addition, phytoene synthase and phytoene dehydrogenase are two key enzymes in the biosynthesis of carotenoids, but these two key enzymes were not found in this study. Furthermore, Yang et al. (2016) had not found these two key enzymes in *C. militaris*, and they believed that the biosynthetic pathway of carotenoids between *C. militaris* and *Neurospora crassa* were totally different. Based on the above, we found that the biosynthetic pathway of carotenoids in *C. militaris* might be different from the biosynthetic pathway of carotenoids in the KEGG database.

Since the biosynthetic pathway of carotenoids in *C. militaris* is unknown, Wang et al. (2018) proposed a putative carotenoid biosynthetic pathway for *C. militaris*. In this putative carotenoid biosynthetic pathway, there were 11 genes involved (CCM_05367, CCM_07659, CCM_00310, CCM_04994, CCM_07937, CCM_05380, CCM_08582, CCM_03203, CCM_03697, CCM_06728, and CCM_09155) (Figure 5). The expression levels of these 11 genes were not significantly different between mycelia 498D (cultured under dark conditions) and mycelia 498L (cultured under light exposure conditions) (Wang et al., 2018). In this study, except for the CCM_03697 gene, the expression levels of the other 10 genes were also not significantly different between mycelia CM10_D and mycelia CM10_L. Hence, the functions of these 11 genes need to be identified.

Since the DEGs involved in carotenoid biosynthesis of *C. militaris* were not found by KEGG pathway analysis, the function of the only terpenoid synthase gene *Cmtns* in DEGs was studied. Although the fruiting bodies produced by the CM10, Δ*Cmtns*, and Δ*Cmtns*-c strains all showed orange color, the carotenoid content of the Δ*Cmtns* strain was significantly lower than that of the CM10 strain, which indicated that the deletion of the *Cmtns* gene could decrease the carotenoid production of *C. militaris*. In addition, we found that the deletion of the *Cmtns* gene also reduced the size of *C. militaris* fruiting bodies, which suggested that the *Cmtns* gene might be a multifunctional gene. These results were also confirmed by the complementation experiment of the *Cmtns* gene.

Because the biosynthetic pathway of carotenoids in *C. militaris* is unknown, and there are few reports on the genes involved in its biosynthesis. Therefore, we believe that the following work will be helpful to clarify the biosynthetic pathway of carotenoids in *C. militaris*: (1) purifying and identifying carotenoids of *C. militaris*; (2) identifying the function of genes involved in the biosynthesis of carotenoids in *C. militaris*; (3) because degeneration of *C. militaris* can lead to a decrease in the content of carotenoids (Sun et al., 2017), elucidating the molecular mechanism of *C. militaris* degeneration will be helpful to reveal the biosynthesis mechanism of *C. militaris* carotenoids; and (4) analyzing the transcriptome of *C. militaris* cultured under a light pulse or a shorter light exposure condition will facilitate the identification of genes involved in biosynthesis of carotenoids (Navarro et al., 2001; Zhang et al., 2016). The comparative transcriptome analysis used in this study laid the foundation for identifying genes involved in the biosynthesis of carotenoids in *C. militaris* and revealing its biosynthetic pathway. In addition, the successful application of the CRISPR-Cas9 gene disruption system in *C. militaris* will accelerate the identification of gene function of *C. militaris* (Chen et al., 2018).

## Conclusion

*Cordyceps militaris* mycelia CM10_D cultured under dark conditions was pure white, while *C. militaris* mycelia CM10_L cultured under light exposure conditions was orange. Comparative transcriptome analysis revealed that there were 1722 DEGs between mycelia CM10_D and mycelia CM10_L. KEGG pathway analysis indicated that the CCM_06728 and CCM_09155 genes might be involved in the biosynthesis of carotenoids in *C. militaris*. What’s more, we identified for the first time that the *Cmtns* gene significantly affected the biosynthesis of carotenoids in *C. militaris*. The results will provide a basis for further research on the biosynthetic pathway of carotenoids in *C. militaris*.

## Data Availability

The datasets generated for this study can be found in the clean reads of the *Cordyceps militaris* transcriptome which has been deposited in NCBI Sequence Read Archive (SRA) under the BioProject accession number PRJNA541819.

## Author Contributions

J-FL and L-QG conceived and designed the experiment. YZ cultured *C. militaris* and performed the transcriptome sequencing. H-BT and Z-WY transformed the *Cmtns* gene into the genome of the Δ*Cmtns* strain. TW constructed the vector pCAMBIA0390-Ben-Comtns and determined the carotenoid content. H-WL analyzed the transcriptome data, identified complementary transformants, and wrote the manuscript. All authors reviewed and approved the submitted version of the manuscript.

## Conflict of Interest Statement

The authors declare that the research was conducted in the absence of any commercial or financial relationships that could be construed as a potential conflict of interest.

## Footnotes

1 http://geneontology.org/

2 https://www.genome.jp/kegg/
