# Supplementary material for: Transcriptome Analysis of Cordyceps militaris Reveals Genes Associated With Carotenoid Synthesis and Identification of the Function of the Cmtns Gene
Source: Front Microbiol. 2019 Sep 10;10:2105. doi: 10.3389/fmicb.2019.02105 (PMC6746990; doi:10.3389/fmicb.2019.02105)
Supplement: Supplementary file 1 [file Data_Sheet_1.docx]

**Supplementary Material**

**Transcriptome Analysis of *Cordyceps militaris* Reveals Genes Associated with Carotenoid Synthesis and Identification of the Function of the *Cmtns* Gene**

Hai-Wei Lou^1,2^, Yu Zhao^2^, Hong-Biao Tang^1^, Zhi-Wei Ye^1^, Tao Wei^1^, Jun-Fang Lin^1,3^* and Li-Qiong Guo^1,3^*

^1^Department of Bioengineering, College of Food Science, South China Agricultural University, Guangzhou, China

^2^College of Food Science and Technology, Henan University of Technology, Zhengzhou, China

^3^Research Center for Micro-Ecological Agent Engineering and Technology of Guangdong Province, Guangzhou, China

**Table S1 |** Oligonucleotide primer sequences used in this study.

| Primer Name | Primer Sequence (5' to 3') |
| --- | --- |
| *tef1*-F | GTCAAGGAAATCCGTCGTGGTAA |
| *tef1*-R | GCAGGCGATGTGAGCAGTGTG |
| 4119-F | CTTCCAAGCAAGCAGAGCAAT |
| 4119-R | GTTCAAGTACGCCGAACCATC |
| 5246-F | GGTCACCATCGCTTCTATTGCC |
| 5246-R | CCTTTTGCACGCTGATAAGCAC |
| 6728-F | CTACCGATTATCAACCCACGAA |
| 6728-R | TTGACCAGACAGTCCAAGAACG |
| 8263-F | ATGCTACCCAACGAAGAAGAGTT |
| 8263-R | AGGTGATAAAGGCAATGTCCAA |
| 9155-F | CATTCTCCCCTTTGACACGCT |
| 9155-R | GATGTGACGTTGAGGAGCACTTT |
| CmtnsC-F | CCTGTGCTCTGTCTCGGTATCATGCTCTGT |
| CmtnsC-R | GTGGAGATGCACCGATGTAGAAAGTCGTAGAT |
| JCBen-F | GGTGCTGCTTTCTGGTACGTCGTCAT |
| JCBen-R | GTCTCGTCGGAGTTCTCAACGAGCTGAT |

**Table S2 |** Summary of gene expression.

| Features | CM10_D | CM10_L |
| --- | --- | --- |
| Total clean reads | 29635460 | 30261506 |
| Total mapping ratio | 64.56% | 62.34% |
| Uniquely mapping ratio * | 40.38% | 38.84% |
| Total gene number | 8793 | 8807 |
| Known gene number | 8655 | 8670 |
| Novel gene number | 138 | 137 |
| Total transcript number | 10761 | 10842 |
| Known transcript number | 7156 | 7251 |
| Novel transcript number | 3605 | 3591 |
| Average coverage of transcripts | 1778 | 1740 |

* Uniquely mapping: Reads that map to only one location of reference, called uniquely mapping.


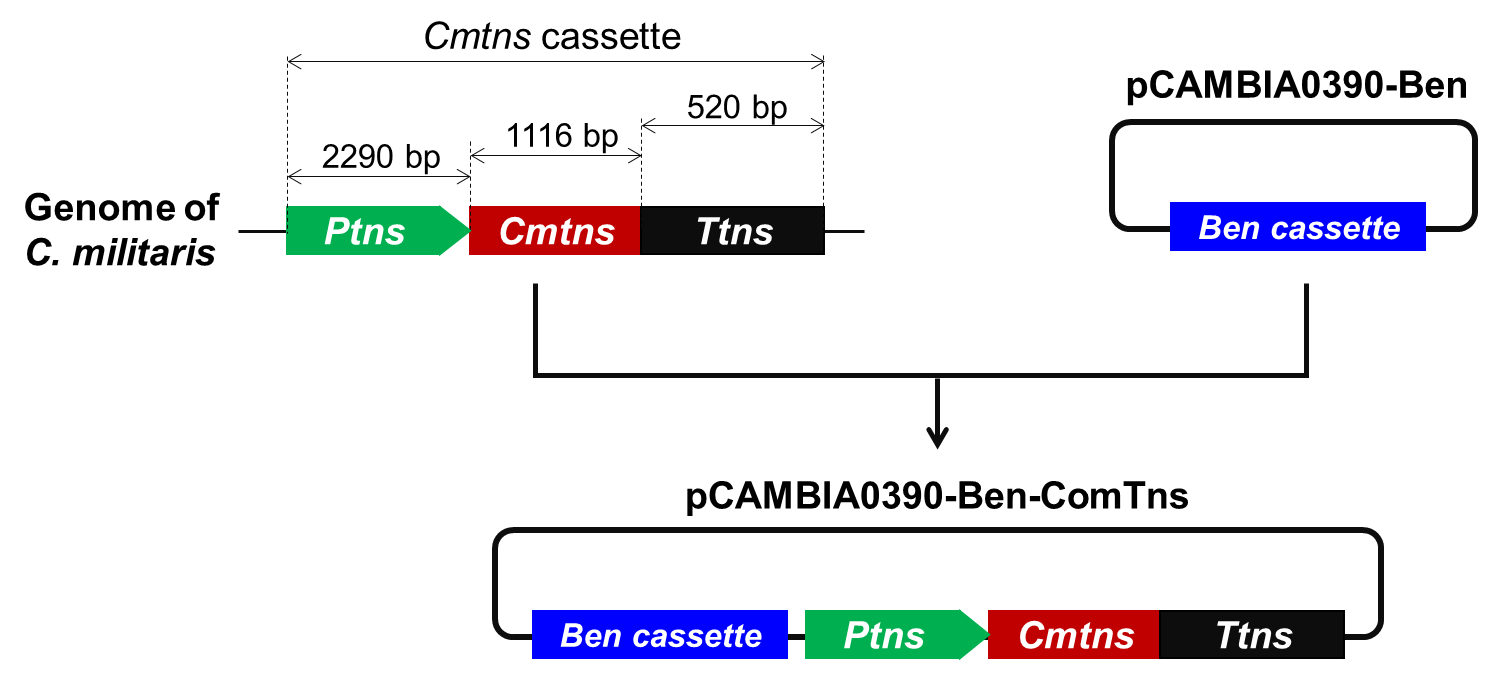


**Figure S1** **|** Construction of plasmid pCAMBIA0390-Ben-Comtns. *Ptns*, promoter of the *Cmtns* gene; *Ttns*, terminator of the *Cmtns* gene.


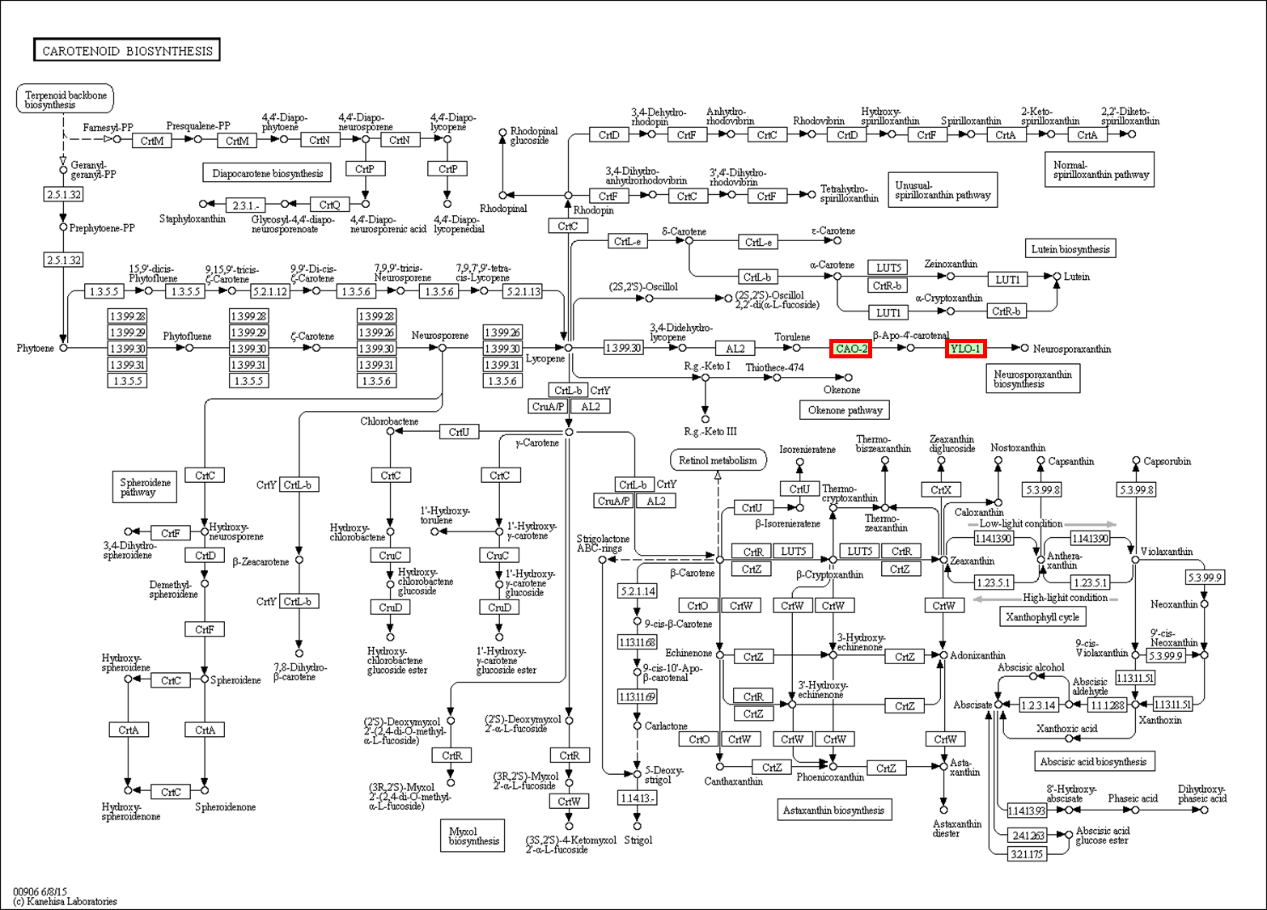


**Figure S2 |** Distribution of *C. militaris* genes CCM_06728 (CAO-2) and CCM_09155 (YLO-1) in carotenoid biosynthetic pathway derived from the KEGG pathway database.
